# Neighborhood Deprivation and Racial Disparities in Heart Failure Outcomes: A Counterfactual Approach

**DOI:** 10.1016/j.jacadv.2025.101808

**Published:** 2025-06-25

**Authors:** Wenxi Huang, Yuru Zhu, Stephen E. Kimmel, Mustafa M. Ahmed, Steven M. Smith, Yao An Lee, Carl Yang, Jiang Bian, Yong Chen, Jingchuan Guo

**Affiliations:** a Department of Pharmaceutical Outcomes and Policy, College of Pharmacy, University of Florida, Gainesville, Florida, USA; b Department of Biostatistics, Epidemiology and Informatics, Perelman School of Medicine, University of Pennsylvania, Philadelphia, Pennsylvania, USA; c Department of Epidemiology, College of Public Health and Health Professions & College of Medicine, University of Florida, Gainesville, Florida, USA; d Division of Cardiovascular Medicine, Department of Medicine, College of Medicine, University of Florida, Gainesville, Florida, USA; e Department of Computer Science, Emory College of Arts and Sciences, Emory University, Atlanta, Georgia, USA; f Department of Biostatistics and Health Data Science, School of Medicine, Indiana University, Indianapolis, Indiana, USA; g Regenstrief Institute, Indianapolis, Indiana, USA; h Indiana University Health, Indianapolis, Indiana, USA

**Keywords:** Area Deprivation Index, heart failure, hospital readmission, neighborhood deprivation, racial disparities

## Abstract

**Background:**

Heart failure (HF) is a major contributor to hospitalizations and mortality in the United States, with significant racial disparities in care access and clinical outcomes. Social determinants of health (SDoH) play a critical role in shaping these disparities.

**Objectives:**

This study aimed to assess the impact of neighborhood deprivation on racial disparities in HF outcomes and quantify the changes in adverse outcomes if non-Hispanic Black (NHB) patients resided in neighborhoods with SDoH level equal to those of non-Hispanic White (NHW) patients.

**Methods:**

We conducted a retrospective cohort study using electronic health records from the University of Florida, including adults hospitalized for HF between 2016 and 2021. SDoH level was measured using the Area Deprivation Index (ADI). The primary outcome was a composite measure of 1-year readmission and all-cause mortality. A counterfactual framework was applied to estimate how NHB patient outcomes might change if they lived in neighborhoods with ADI distributions equivalent to NHW patients.

**Results:**

Among 42,279 patients (mean age 65 ± 14.3 years; 48% women), NHB patients had more 1-year composite outcomes (32.92%) compared to NHW patients (27.69%). Adjusted analyses showed NHB patients had a higher risk of readmission or mortality (aOR: 1.101; 95% CI: 1.063-1.139). Counterfactual modeling showed that if NHB patients resided in neighborhoods with ADI distributions same as NHW patients, their outcome rate would decrease by 1.31% (95% CI: 1.309%-1.311%).

**Conclusions:**

This study highlights racial disparities in HF outcomes attributed to neighborhood deprivation. Improving socioeconomic conditions in deprived neighborhoods could mitigate disparities in HF.

Heart failure (HF) remains one of the leading causes of morbidity and mortality worldwide, placing a substantial burden on health care systems and patients' quality of life.^1^^,^^2^ Despite advances in medical management and technology, HF patients often face high rates of hospital readmission and mortality.^3^ In the United States, the number of people hospitalized with HF increased from one million in 2008 to 1.3 million in 2018.^4^ The HF readmission rate within 1 year is approximately 55% in adult hospitalization for HF (HHF) patients, and the 1-year mortality rate is about 25%.^5^, ^6^, ^7^ These adverse outcomes are influenced by a complex interplay of clinical, demographic, and socioeconomic factors.^8^

Racial disparities in health outcomes are well-documented, with minority populations often experiencing worse health outcomes due to a combination of socioeconomic disadvantages, health care access issues, and potential biases in medical care.^9^ For example, non-Hispanic Black (NHB) patients have been shown to have higher rates of hospital readmission and mortality compared to non-Hispanic White (NHW) patients, largely due to disparities in socioeconomic status (SES), health care access, and quality of care.^1^ Social determinants of health (SDoH) have been increasingly recognized as critical determinants of racial disparities in HF health outcomes.^10^ Several studies have indicated that lower SES is associated with higher readmission rates and mortality among HF patients.^11^^,^^12^ For instance, patients with lower income levels often face barriers to accessing health care, adhering to treatment plans, and maintaining healthy lifestyles, which can exacerbate HF symptoms and lead to worse outcomes.^10^

While previous studies have explored individual or joined aspects of SDoH such as income, education, and access to health care, none have employed a counterfactual simulation framework to specifically quantify the impact of neighborhood deprivation on racial disparities in HF outcomes by modeling the effect of hypothetically placing one group in the same SDoH environment as another.^8^, ^9^, ^10^ In this study, we aimed to quantify the impact of neighborhood deprivation, as measured by the Area Deprivation Index (ADI), on racial disparities in HF adverse outcomes, specifically focusing on a composite primary outcome of 1-year HF readmission or all-cause mortality between NHW and NHB patients in a real-world cohort from the University of Florida (UF) Health electronic health records (EHRs). We employed a counterfactual modeling approach to the potential reduction in HF-related adverse outcomes if NHB patients resided in neighborhoods with ADI distributions same as those of NHW patients.^13^^,^^14^ This method allows us to isolate the effect of neighborhood deprivation on health outcomes and quantify the potential reduction in disparities if socioeconomic conditions were equalized.

## Methods

### Data source and study population

We conducted a retrospective cohort study utilizing EHRs from the UF Integrated Data Repository (IDR), covering the period from 2016 to 2021, with patient follow-up extending through 2022.^15^ The IDR includes comprehensive patient-level data such as diagnoses, procedures, medications, and demographic information for patients treated within UF Health's clinical practice. Institutional Review Board approval was obtained (UF IRB reference # IRB202201080) before conducting the study.

The study cohort comprised patients aged ≥18 who experienced at least one HF hospitalization between January 1, 2016, and January 31, 2021. These patients were followed for up to 1 year, extending through 2022. HF hospitalization was defined as a primary admission diagnosis of HF, identified using International Classification of Diseases (ICD)-10 codes (I50.x, I11.0, I13.0, I13.2, I97.13, I09.81), which have previously been validated against discharge summaries or medical records, yielding a positive predictive value of 100% (95% CI: 92.9-100).^16^ The index date for each patient was defined as the date of their first recorded HHF during the study period. Each patient was followed for 1 year postindex HHF. We excluded patients without geographic information (eg, Federal Information Processing Standard code), those who had undergone heart transplants or had a left ventricular assist device, and patients whose self-reported race was other than NHB or NHW.

### Study outcome

The primary outcome was a composite measure of HF readmission or all-cause mortality, measured within 1 year of follow-up since index date. HF readmissions were identified using the same ICD-10-Clinical Modification (CM) codes detailed in the Data Source and Study Population section.^16^ All-cause mortality was determined through death records in the IDR, which is linked to the National Death Index.

#### Exposure of interest

The ADI is a composite measure that captures contextual SDoH using a variety of indicators such as income, education, employment, and housing quality and is aggregated in a 5-year moving average.^17^ The ADI provides a robust means to assess the socioeconomic context of different geographic areas, enabling researchers and policymakers to identify and address disparities in health outcomes linked to socioeconomic deprivation.^12^

In this study, we selected the 2020 ADI, which covers data from 2016 to 2020, aligning closely with our study period. The ADI is a factor-based index that utilizes 17 U.S. Census indicators, including measures of poverty, education, housing quality, and employment, to assess and rank the socioeconomic contextual disadvantage of neighborhoods.^17^

We conducted a spatiotemporal linkage of the ADI national percentile rankings to our patient data using the geographic federal information processing standard code at the county level based on the patient's home address. The county-level ADI was calculated by taking the median block group ADI ranking for all patients within a county.^18^ In order to quantify the impact of association between neighborhood deprivation and adverse health outcomes in HF patients, we categorized counties into 3 groups for analysis. The three-group categorization, based on the quintile distribution, comprises low (bottom 20%), medium (21-80%), and high (top 20%) levels of ADI. This categorization was adapted from quintile-based approaches utilized in prior research, which commonly distinguish neighborhoods into least deprived and most deprived segments to investigate associations with health outcomes.^19^, ^20^, ^21^

#### Covariates

Covariates were assessed in the 3 years prior to the index date. Covariates were selected based on prior research and clinical expertise and included age, sex, and comorbidities.^22^, ^23^, ^24^, ^25^, ^26^, ^27^ Comorbidities included HF with preserved ejection fraction (HFpEF), chronic obstructive pulmonary disease (COPD), myocardial infarction, anemia, implantable cardioverter-defibrillator and/or cardiac resynchronization therapy(ICD and/or CRT), diabetes, and cancer. These conditions were identified using ICD-9-CM or ICD-10-CM diagnosis and procedure codes (refer to Supplemental Table 1).^28^, ^29^, ^30^, ^31^, ^32^ We developed a Directed Acyclic Graph informed by published literature and expert consultation, depicting causal assumptions regarding the interplay between neighborhood socioeconomic deprivation, race, and clinical comorbidities (Supplemental Figure 1).

### Statistical analysis

We reported descriptive statistics of baseline patient characteristics, including number and percentage for categorical variables and means and SDs or medians and IQRs for continuous variables. We then examined the distribution of ADI across racial groups. We applied logistic regression models to access the association between racial disparities in HF outcomes with an adjustment of age, sex, history of HFpEF, history of COPD, history of myocardial infarction, history of anemia, history of ICD and/or CRT-D, history of diabetes, history of cancer, and ADI. To better understand the interplay between race/ethnicity and neighborhood deprivation, we performed additional analyses. First, we examined the association between race/ethnicity and HF outcomes in a model without adjusting for ADI. In this analysis, if the variable Black race was significantly associated with the outcome, it indicated that ethnicity independently influences HF outcomes. We then conducted a second model examining the effect of ADI on outcomes, adjusting for relevant clinical and demographic variables but not race both race and ADI.

Next, we applied our recently developed counterfactual framework (dGEM-disparity: decentralized algorithm for generalized linear mixed effect model for disparity quantification) to quantify the effect of neighborhood deprivation on racial disparities in adverse health outcomes of HF patients.^14^^,^^33^ In this dGEM framework, our model included common (fixed) effects representing patient-level factors such as demographic characteristics (race, age, and sex), health care utilization (insurance status and length of inpatient stay), and clinical variables (HFpEF, COPD, mechanical ventilation, myocardial infarction, anemia, and cancer). These variables were chosen for their consistent and systematic impact on outcomes across the study population. Additionally, random effects were introduced to capture regional variability in HF outcomes attributable to local differences in ADI. Specifically, random effects were modeled at 3 categorical levels to account for unobserved geographical heterogeneity beyond the patient-level factors. This approach enabled us to quantify both the common effects across all patients and the variation in outcomes across regions.

Specifically, we simulated the outcomes for NHB patients as if they resided in neighborhoods with ADI distributions same as those of NHW patients. The counterfactual model was built using a federated algorithm for generalized linear mixed models (Fed-GLMM), which enabled joint modeling across multiple sites by leveraging local computations and sharing aggregated data.^14^ This method ensured robust model accuracy while maintaining data privacy across the sites.^34^

In the simulation, each NHB patient was assigned to one of 3 ADI regions using a multinomial distribution with the probabilities being the proportions of NHW patients distributed over all 3 ADI regions. We then estimated the individual risk of 1-year readmission or mortality for NHB patients under this counterfactual scenario. This process was repeated 400 times to provide estimates of uncertainty. Further methodological details are provided in the supplementary material.

In addition to our primary analysis of the composite outcome, we performed a sensitivity analysis focusing on 1-year readmission outcomes. Using the same counterfactual modeling framework, we estimated the 1-year readmission rate for Black patients under the scenario in which they resided in neighborhoods with ADI same as those of White patients.

All statistical tests were 2-sided, with statistical significance set at *P* < 0.05. All analyses were conducted using R, version 4.3.1 (R Foundation for Statistical Computing).

## Results

### Baseline characteristic of the study cohort

**Figure 1:**
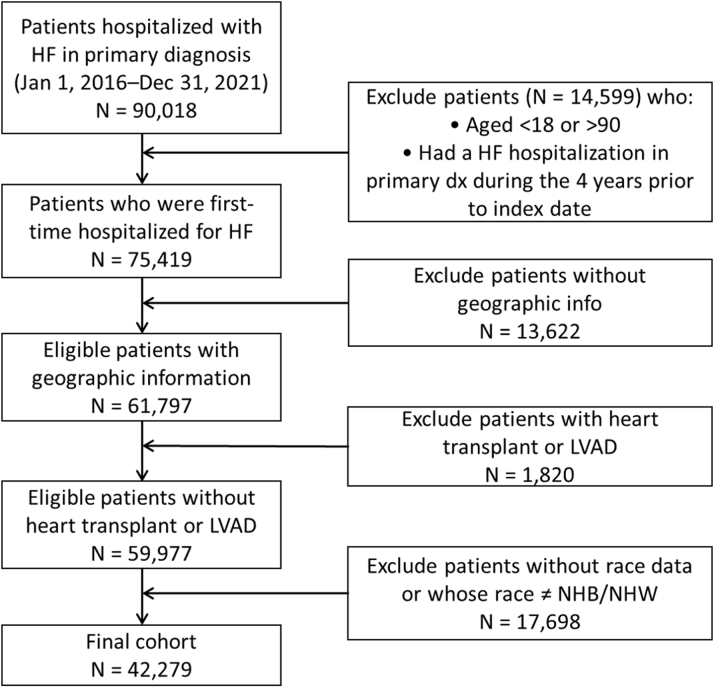
**Patient Flowchart** HF = heart failure; LVAD = left ventricular assist devices; NHB = non-Hispanic Black; NHW = non-Hispanic White.

**Figure 2:**
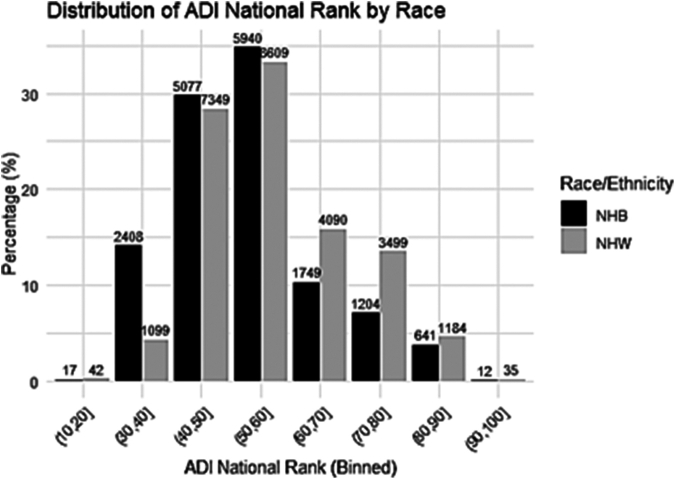
**The Distribution of ADI by Race** ADI = Area Deprivation Index; other abbreviations as in Figure 1.

**Figure 3:**
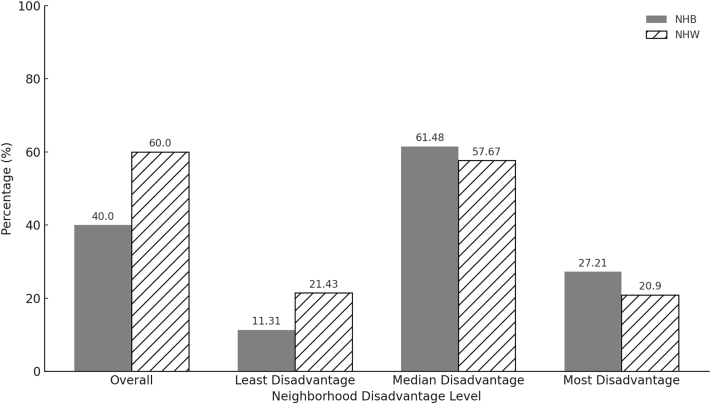
**Distribution of NHB and NHW Individuals Across 3 Levels of Disadvantage** Abbreviations as in Figure 1.

Our study cohort comprised a total of 42,279 eligible patients with a mean age of 65 ± 14.3 years (Figure 1). Of these, 48% were female, 60% were NHW, 40% were NHB, and 64% were uninsured. Additionally, 53% had a history HFpEF, 38% had COPD, 16% had experienced a myocardial infarction, 48% had anemia, 8% had an ICD and/or CRT, 50% had diabetes, 12% had cancer, 9% had undergone mechanical ventilation, and 82% had an initial HF hospitalization stay of ≤4 days (Table 1). When analyzing neighborhood socioeconomic status using the ADI, we observed significant differences between racial groups (Figure 2). Of the patients living in the least disadvantaged neighborhoods, 11% are NHB compared to 21% of NHW patients. In contrast, 27% of NHB patients live in the most disadvantaged neighborhoods, while only 21% of NHW patients reside in the same deprived areas (Figure 3).

**Table 1:** Patient Baseline Characteristics, by ADI

	Overall (N = 42,279)	Least Disadvantage (n = 7,365)	Median Disadvantage (n = 25,021)	Most Disadvantage (n = 9,893)	*P* Value^a^
Race/ethnicity					<0.001
NHB	16,766 (40%)	1,897 (26%)	10,307 (41%)	4,562 (46%)	
NHW	25,513 (60%)	5,468 (74%)	14,714 (59%)	5,331 (54%)	
Age, y	64.75 ± 14.27	63.52 ± 14.66	65.22 ± 14.22	64.31 ± 14.03	<0.001
Age group					<0.001
<45	3,759 (8.9%)	449 (6.1%)	2,321 (9.3%)	989 (10.0%)	
45-64	17,168 (41%)	2,443 (33%)	10,350 (41%)	4,375 (44%)	
65-74	9,434 (22%)	1,650 (22%)	5,573 (22%)	2,211 (22%)	
≥75	11,918 (28%)	2,823 (38%)	6,777 (27%)	2,318 (23%)	
Female	20,340 (48%)	3,334 (45%)	12,101 (48%)	4,905 (50%)	<0.001
Insurance					0.084
Medicaid	1,101 (2.6%)	208 (2.8%)	659 (2.6%)	234 (2.4%)	
Medicare	5,648 (13%)	1,006 (14%)	3,353 (13%)	1,289 (13%)	
Missing	6,881 (16%)	1,157 (16%)	4,073 (16%)	1,651 (17%)	
Other	939 (2.2%)	178 (2.4%)	553 (2.2%)	208 (2.1%)	
Private	624 (1.5%)	129 (1.8%)	339 (1.4%)	156 (1.6%)	
Uninsured	27,086 (64%)	4,687 (64%)	16,044 (64%)	6,355 (64%)	
HFpEF	22,267 (53%)	3,910 (53%)	13,196 (53%)	5,161 (52%)	0.50
COPD	16,038 (38%)	2,434 (33%)	9,308 (37%)	4,296 (43%)	<0.001
Myocardial infarction	6,888 (16%)	1,086 (15%)	3,911 (16%)	1,891 (19%)	<0.001
Anemia	20,408 (48%)	3,434 (47%)	12,094 (48%)	4,880 (49%)	0.002
ICD and/or CRT	3,385 (8.0%)	577 (7.8%)	1,984 (7.9%)	824 (8.3%)	0.40
Diabetes	21,153 (50%)	3,346 (45%)	12,590 (50%)	5,217 (53%)	<0.001
Cancer	5,037 (12%)	1,013 (14%)	2,860 (11%)	1,164 (12%)	<0.001
Mechanical ventilation	3,615 (8.6%)	565 (7.7%)	2,207 (8.8%)	843 (8.5%)	0.008
Length of stay (d)					<0.001
1	9,273 (22%)	1,483 (20%)	5,489 (22%)	2,301 (23%)	
2	11,848 (28%)	2,009 (27%)	6,931 (28%)	2,908 (29%)	
3	7,477 (18%)	1,302 (18%)	4,394 (18%)	1,781 (18%)	
4	5,929 (14%)	1,073 (15%)	3,548 (14%)	1,308 (13%)	
5 (+)	7,752 (18%)	1,498 (20%)	4,659 (19%)	1,595 (16%)	
1-year readmission outcome	10,733 (25%)	1,752 (24%)	6,469 (26%)	2,512 (25%)	0.002
1-year composite outcome	12,568 (30%)	2,059 (28%)	7,439 (30%)	3,070 (31%)	<0.001

Values are n (%) or mean ± SD.

ADI = Area Deprivation Index; COPD = chronic obstructive pulmonary disease; CRT = cardiac resynchronization therapy; HFpEF = heart failure with preserved ejection fraction; ICD = implantable cardioverter-defibrillator; NHB = non-Hispanic Black; NHW = non-Hispanic White.

a Pearson's chi-squared test; Kruskal-Wallis rank sum test.

## Association between race/ADI and HF adverse outcomes

We used a random effect logistic regression to test the association between racial disparities or ADI and HF adverse outcomes. For the composite outcome of 1-year HF readmission and mortality, after adjusting for age, sex, history of HFpEF, history of COPD, history of myocardial infarction, history of anemia, history of ICD and/or CRT-D, history of diabetes, history of cancer, and ADI, the adjusted OR for NHB vs NHW patients was 1.313 (95% CI: 1.252-1.377), reflecting a 31.3% increased odds of the composite outcome (Supplemental Table 2).

For the association between ADI and adverse HF outcomes, after adjusting for age, sex, history of HFpEF, COPD, myocardial infarction, anemia, ICD and/or CRT-D, diabetes, cancer, and race, the adjusted OR for living in a medium ADI area or high ADI area, vs a low ADI area, was 1.068 (95% CI: 0.994-1.148), and 1.044 (95% CI: 0.96-1.135), respectively (Supplemental Table 2).

Additionally, in our model without ADI, the association between race and HF outcomes was statistically significant (Black race: OR: 1.314; 95% CI: 1.254-1.378) (Supplemental Table 3). Compared to the first model that included both race and ADI, we observed that race/ethnicity is independently associated with HF outcomes. We also evaluated the effect of neighborhood deprivation by including ADI categories and adjusting for clinical covariates and demographic variables, excluding race. The adjusted OR for medium vs low ADI was 0.968 (95% CI: 0.894-1.048), and for high vs low ADI, it was 0.919 (95% CI: 0.841-1.004) (Supplemental Table 4). These findings suggest that while ADI is associated with HF outcomes, its effect may be modest and potentially influenced by how race and neighborhood factors intersect within the analytic model.

### Counterfactual effect of ADI on racial disparities in HF outcome

**Figure 4:**
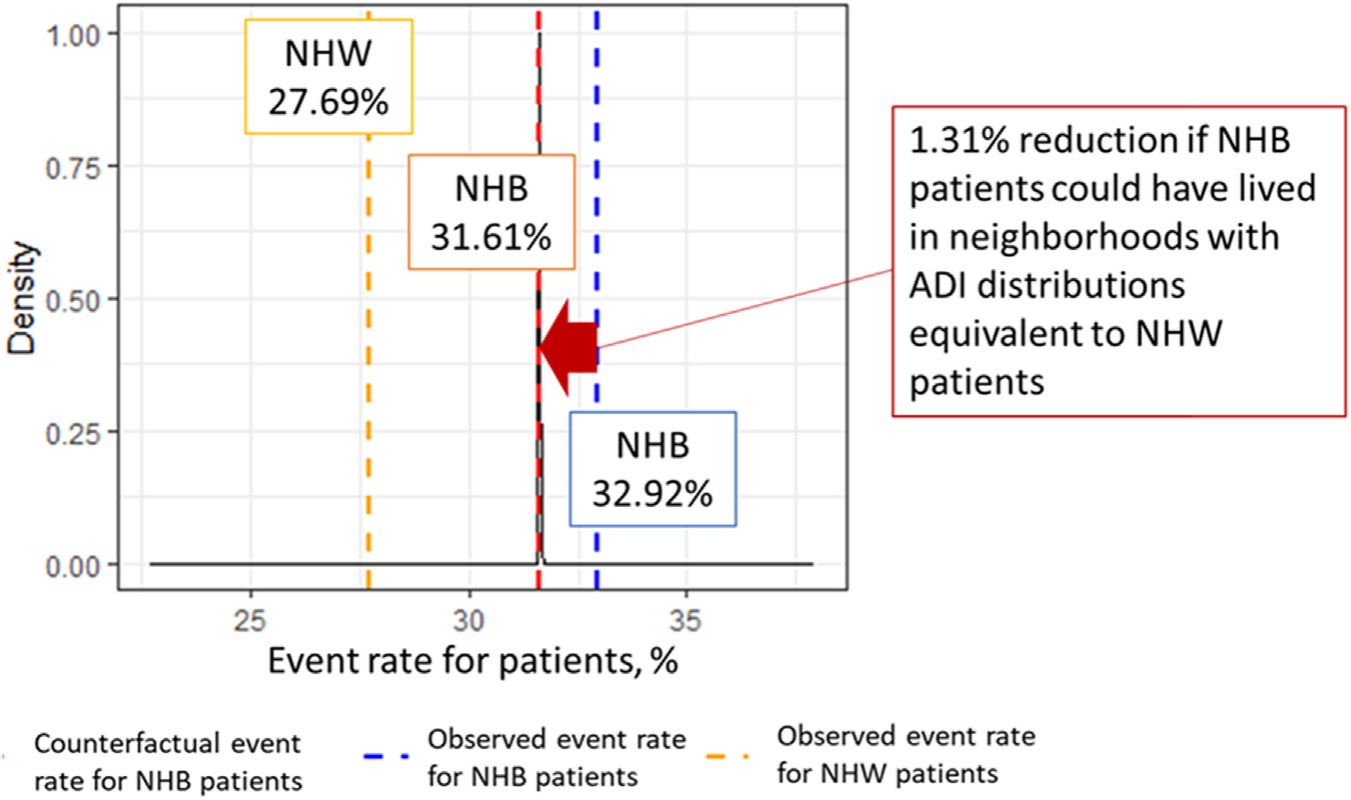
**The Observed 1-Year Readmission/Mortality Event Rate and the Estimated Counterfactual Event Rate** Abbreviations as in Figures 1 and 2.

**Figure 5:**
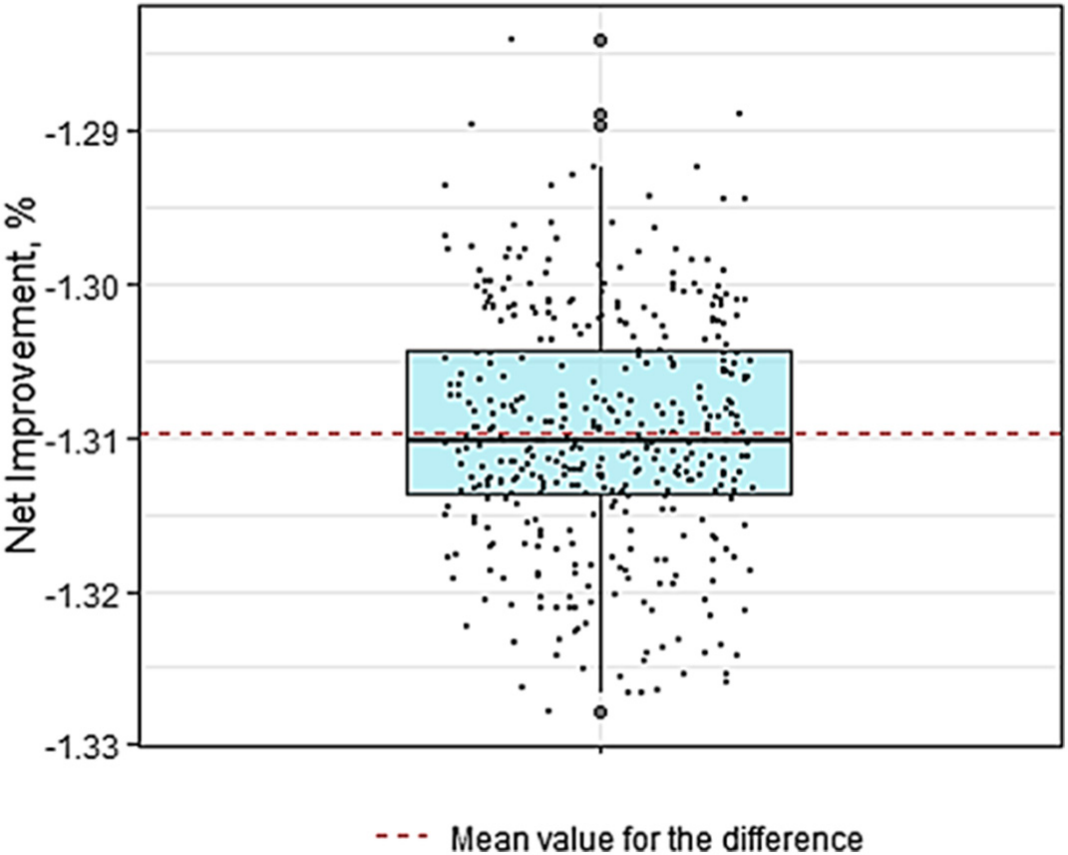
**Distribution of Differences in Event Rates Between Observed and Counterfactual Scenarios** This plot shows the distribution of the net improvement (%) for each of 400 counterfactual simulations.

Figure 4 illustrates the differences between the observed rates and counterfactual estimates, which simulate the potential outcomes if NHB patients had resided in neighborhoods with ADI distributions same as those of NHW patients. After quantifying both the common effects across all patients (Table 2) and the variation in outcomes across regions (Table 3), we conducted 400 simulation iterations to derive counterfactual event rate estimates. Figure 5 presents the distribution of the differences between the observed and estimated event rates across these simulations. The observed 1-year HF readmission or mortality rate for NHW patients was 27.69%, and for NHB patients, it was 32.92%. In the counterfactual scenario—where NHB patients resided in neighborhoods with ADI distributions equivalent to NHW patients—the estimated 1-year HF readmission or mortality rate for NHB decreased by 1.31% (95% CI: 1.309%-1.311%) to 31.61%. This finding indicates that, on average, 131 per 10,000 fewer NHB patients would have experienced either HF readmission or death within 1 year after their initial HF hospitalization if they had lived in neighborhoods with same ADI distributions as NHW patients. The estimated common and random effects from our dGEM analysis are presented in Tables 2 and 3, respectively. Table 2 shows the fixed effects estimates for demographic, health care utilization, and clinical variables, while Table 3 displays the random effects capturing regional variability in HF outcomes.

**Table 2:** Estimated Common Effects With Standard Errors and *P* Values for 1 Year Readmission or Mortality Outcome

	Estimated Log OR	Standard Error	*P* Value
Black race	0.205	0.043	<0.001^a^
Age	−0.005	0.002	0.007^b^
Male	0.141	0.052	0.007^b^
Insurance			
Medicaid	−0.282	0.049	<0.001^a^
Medicare	0.026	0.110	0.817
Missing	0.083	0.093	0.373
Uninsured	0.012	0.089	0.896
Other	−0.016	0.114	0.890
Comorbidities			
HFpEF	−0.143	0.022	<0.001^a^
COPD	0.198	0.023	<0.001^a^
Mechanical ventilation	−0.282	0.049	<0.001^a^
Myocardial infarction	0.213	0.029	<0.001^a^
Anemia	0.159	0.034	<0.001^a^
Cancer	0.072	0.033	0.032^c^
Length of initial inpatient stay			
2	−0.038	0.031	0.213
3	−0.009	0.034	0.787
4	0.052	0.036	0.153
5	0.096	0.046	0.036^c^

Abbreviations as in Table 1.

a Statistical significance: *P* < 0.05.

b Statistical significance: *P* < 0.01.

c Statistical significance: *P* < 0.001.

**Table 3:** Random Effects for 3 ADI Level Regions for 1 Year Readmission or Mortality Outcome

Site 1	Site 2	Site 3
−0.588	−0.705	−0.611

Abbreviation as in Table 1.

#### Model evaluation

Key model assumptions and performance were thoroughly evaluated. While logistic regression does not require normally distributed residuals, we confirmed the linearity of the logit for continuous predictors. Multicollinearity among the fixed-effect covariates was assessed using variance inflation factors.^35^ Our results showed that the variance inflation factor (1/[2∗Df]) values ranged from approximately 1.002 to 1.080—well below the conventional threshold of 5—indicating negligible collinearity among predictors (Supplemental Table 5).^36^

Model calibration was evaluated using the Hosmer-Lemeshow goodness-of-fit test, and the test did not indicate significant lack of fit.^37^ Model performance was quantified using Nagelkerke's pseudo R^2^, with a marginal pseudo R^2^ (R^2^_m) of 0.0168 and a conditional pseudo R^2^ (R^2^_c) of 0.0409 (Supplemental Table 6). This suggests that the fixed effects account for a modest portion of the variance in the outcome, while the inclusion of random effects at the site level captures additional variability.^38^^,^^39^

Furthermore, residual diagnostics using the DHARMa package indicated no significant overdispersion or systematic biases (Supplemental Figure 2).^40^ Overall, these diagnostics confirm that our model meets the necessary assumptions for reliable inference.

#### Sensitivity analysis

**Figure 6:**
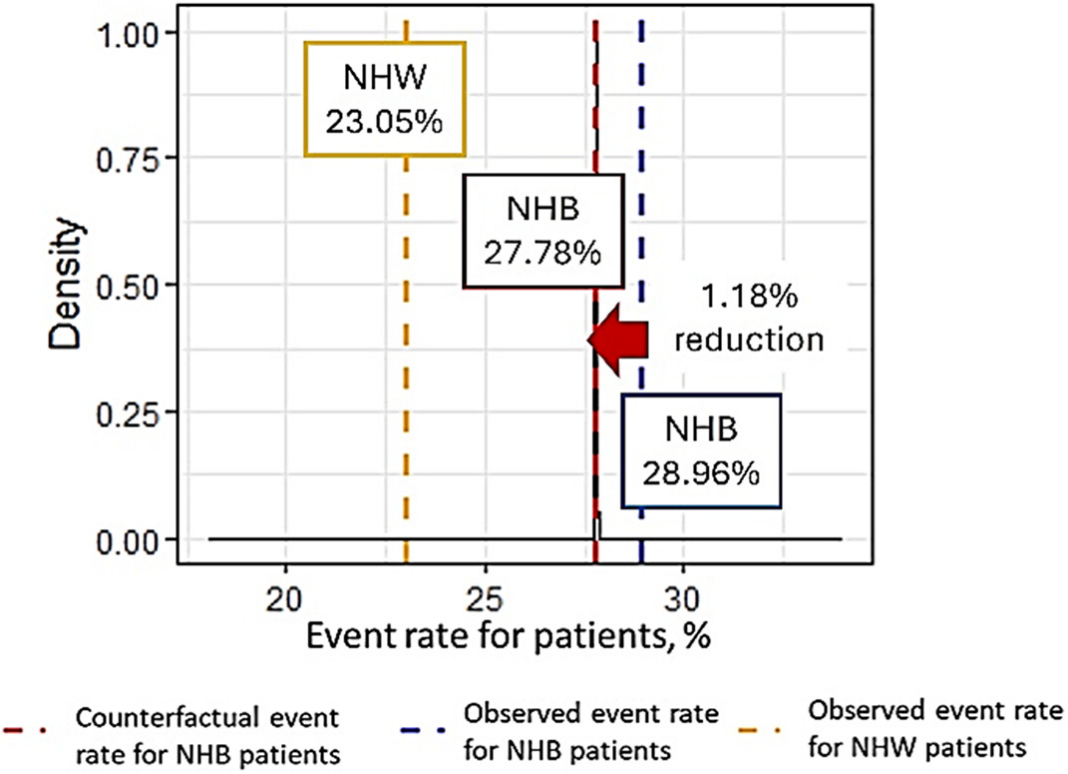
**The Observed 1-Year Readmission Event Rate and the Estimated Counterfactual Event Rate** Abbreviations as in Figure 1.

The sensitivity analysis for 1-year readmission outcomes is summarized in Table 6. The estimated counterfactual 1-year readmission rate for Black patients was 27.78%, compared with an observed rate of 28.96% among Black patients and 23.05% among White patients (Figure 6). This yields a difference of 1.18 percentage points between the counterfactual rate for Black patients and the observed rate for White patients. These findings suggest that, under the counterfactual scenario, aligning the ADI conditions of Black patients with those of White patients would result in a modest reduction in readmission rates.

## Discussion

In this study, we applied a novel counterfactual framework to quantify the impact of neighborhood deprivation on racial disparities in HF outcomes. After adjusting for key clinical factors that may impact HF-related outcomes, we observed that NHB patients had a 31.3% increased odds of experiencing the composite outcome of 1-year HF readmission or mortality compared to NHW patients. Our counterfactual analysis, which simulated the hypothetical scenario in which NHB patients resided in neighborhoods with ADI distributions the same as those of NHW patients, demonstrated a small but meaningful reduction in adverse outcomes. Specifically, the HF readmission or mortality rate decreased by 1.31%, equating to 131 fewer adverse events per 10,000 NHB patients (Central Illustration). While this reduction represents a modest impact relative to the overall risk of HF readmission or mortality, it underscores the broader impact of SDoH, particularly neighborhood deprivation, in shaping racial disparities in HF outcomes.

**Central Illustration:**
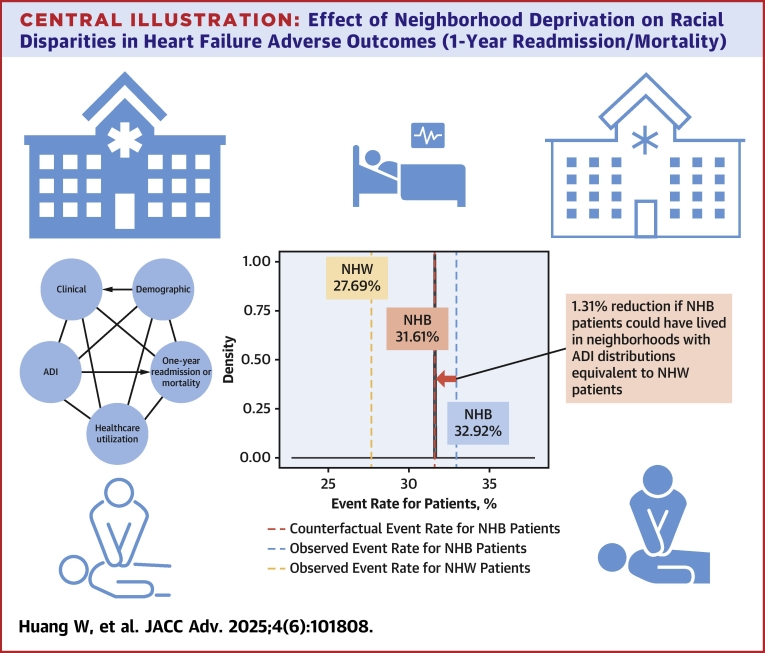
**Effect of Neighborhood Deprivation on Racial Disparities in Heart Failure Adverse Outcomes (1-Year Readmission/Mortality)** Our study cohort comprises 42,279 patients hospitalized with heart failure from the University of Florida Health electronic health records between 2016 and 2021, with a mean age of approximately 65 years, 48% female, 60% non-Hispanic White, and 40% non-Hispanic Black. For 1 year readmission or mortality outcome, the observed event rates were 27.69% for NHW patients (displayed in orange) and 32.94% for NHB patients (displayed in blue). The estimated counterfactual event rate for NHB patients is 31.61% (displayed in red), representing the scenario if NHB patients had resided in neighborhoods with ADI distributions same as those of NHW patients. Abbreviations as in Figures 1 and 2.

Despite adjusting for ADI, we observed a persistent racial disparity in HF outcomes, with NHB patients maintaining nearly a 4% higher absolute risk compared to NHW patients (31.61% vs 27.69%). This residual disparity suggests the presence of additional unmeasured factors beyond neighborhood deprivation alone.

Our sensitivity analysis focusing on 1-year readmission outcomes further supports the robustness of our counterfactual modeling. While the estimated counterfactual rate for Black patients (27.78%) indicates a modest improvement compared to the observed rate (28.96%), the persistence of a 1.18 percentage point difference relative to White patients (23.05%) underscores that neighborhood deprivation is only one factor contributing to disparities in HF outcomes.

Potential contributors include structural racism, implicit biases within health care systems, and differential access to high-quality medical care.^41^^,^^42^ Structural racism can perpetuate inequities through mechanisms such as residential segregation, disparities in employment opportunities, differential exposure to environmental stressors, and systemic barriers to health care access.^41^^,^^42^ Furthermore, individual-level socioeconomic status, health care literacy, social support networks, and genetic predispositions might also influence this remaining disparity.^43^^,^^44^ Future research should explicitly incorporate both structural and individual-level factors to comprehensively characterize and address the multifaceted drivers of racial disparities in HF outcomes.

Our findings are consistent with previous studies that have demonstrated the significant role socioeconomic deprivation plays in poor HF outcomes and the amplification of racial disparities in disadvantaged neighborhoods. Consistent with previous research, we found that NHB patients tend to experience worse outcomes after HF hospitalization.^45^^,^^46^ Additionally, our results support existing evidence that greater neighborhood deprivation is associated with worsen HF outcomes.^47^^,^^48^ However, our study goes a step further by not only confirming these associations but also quantifying the specific effect of neighborhood deprivation on such racial disparities, as measured by the ADI. By simulating the redistribution of NHB patients into neighborhoods with more favorable ADI scores, we were able to estimate the contribution of neighborhood socioeconomic conditions to racial disparities in HF outcomes. The reduction in readmission and mortality rates in this counterfactual scenario underscores the importance of addressing neighborhood-level deprivation to mitigate these disparities.

Several mechanisms may explain the observed association between race, ADI, and adverse HF outcome. ADI is a composite measure of neighborhood socioeconomic disadvantage, encompassing income, education, employment, and housing quality.^17^ These factors collectively shape health outcomes by influencing health care access, health literacy, and exposure to environmental stressors that contribute to disease progression.^12^ For example, individuals in high-ADI neighborhoods often face significant barriers, including limited access to primary and specialty care, medication unaffordability, food insecurity, and substandard housing conditions, and these disparities can exacerbate existing clinical risks in HF patients, leading to higher rates of readmission and mortality.^49^, ^50^, ^51^, ^52^

Our study has several strengths. This study is reported in accordance with the STROBE (Strengthening the Reporting of Observational Studies in Epidemiology) guidelines for cohort studies (see Supplemental Table 7). We used population-based data from a large geographic area, and we deterministically linked comprehensive health care administrative data using unique numeric identifiers. Individuals with HF hospitalization were identified through a validated administrative data algorithm. Given the complex interplay between socioeconomic factors and HF outcomes, our study leveraged a robust methodological approach to accurately quantify these disparities. The use of Fed-GLMM allowed us to appropriately account for hierarchical data structures, where patients are nested within neighborhoods that share common socioeconomic and health care access characteristics.^14^ This approach accounted for clustering effects and case mix variations across different levels of social deprivation, reducing biases that may arise from treating all observations as independent. The iterative process of refining model parameters through local computations and shared aggregated data ensured the accuracy and reliability of our findings. In this project, the dGEM model accounted for variations in case mix between different social deprivation level sites, enabling the analysis of large and heterogeneous data sets in multisite studies while adjusting for between-site differences.

Our study highlights the necessity of targeted interventions that address SDoH, particularly in deprived neighborhoods with predominantly racial/ethnic minority and socioeconomically disadvantaged populations. Understanding how SDoH interact with racial disparities is crucial for developing targeted interventions, including policies that improve health care accessibility in disadvantaged areas and community-based programs aimed at enhancing chronic disease management. Structural interventions that address social inequities at the neighborhood level are essential to achieving more equitable HF outcomes. Future research, such as policy evaluations, is needed to assess the real-world impact of interventions aimed at mitigating neighborhood deprivation and reducing HF disparities.

### Study Limitations

Our study is subject to several limitations. First, although we used counterfactual modeling to estimate the potential impact of neighborhood deprivation on outcomes, there remains unmeasured confounders that could influence outcomes, such as health care access or social support. Second, our study was based on data from a single source predominately including residents from Florida, which may limit the generalizability of our findings to other regions with different demographic compositions. Third, standardized HF severity classifications such as the NYHA functional classification or the American Heart Association stages of HF were not available in our data set. However, we attempted to account for HF severity by adjusting for clinically relevant proxies, including the presence of ICD and/or CRT-D, length of initial hospitalization stay, and comorbid conditions such as COPD, myocardial infarction, anemia, diabetes, and cancer. While these proxies provide valuable insight into HF severity, the absence of standardized classifications remains a limitation. Fourth, a potential limitation of this study is the reliance on diagnosis codes to define HF readmissions. Although we restricted readmissions to cases where HF was the primary diagnosis, there remains the possibility of misclassification due to human error. However, we assume that any such misclassification would occur at random rather than systematically biasing the results. Fifth, our analyses confirm that race/ethnicity is significantly associated with HF outcomes and ADI. While our models suggest that ADI may have a modest effect on outcomes, this effect was not statistically significant when race was included. It is plausible that the impact of ADI on outcomes may be mediated by race/ethnicity. However, a formal mediation analysis was not performed in this study. The complex interplay between social determinants and race in our data set warrants a more extensive investigation that goes beyond the scope of the present study. Future research should explore this mediation pathway to further elucidate the complex relationships among race, neighborhood socioeconomic conditions, and HF outcomes. Sixth, we applied county-level ADI for our analysis, which may limit our ability to capture finer granular variations in neighborhood deprivation and its effects on HF outcomes. Furthermore, while this study focuses on ADI as a composite measure of contextual SDoH disadvantage, we acknowledge that individual social and environmental factors—such as education, employment, transportation access, health care availability, and neighborhood built environment—also play a critical role in shaping health outcomes. The absence of individual-level SDoH prevents us from accounting for the potential confounding effects of person-level SDoH in the relationship between ADI and HF disparities and outcomes. However, to some extent, contextual-level SDoH and person-level SDoH are correlated, and contextual-level SDoH often serve as proxies for person-level SDoH, particularly when individual-level data are unavailable (as is common in real-world data sets such as EHRs and insurance claims).^53^ Additionally, contextual-level SDoH can capture structural inequities that contribute to health disparities.^54^ Future studies incorporating these specific factors at a finer geographic scale (eg, census tracts) will provide additional insights into the mechanisms driving HF disparities. Future research should explore the relative contributions of both contextual- and person-level SDoH to better understand their interactions and inform targeted interventions. And to further investigate the influence of individual SES components on HF outcomes, we have planned an external exposome-wide association study, which will investigate the SDoH impact on HF outcomes from a different angle by systematically examining a broad range of external environmental exposures beyond ADI.^55^

## Conclusions

This study underscores the influence of neighborhood deprivation on racial disparities in adverse HF outcomes. Our results indicate that addressing socioeconomic conditions in deprived neighborhoods could hypothetically improve health outcomes and promote health equity. Integrating SDoH into HF management strategies could be a critical step toward reducing disparities, and our study highlights the potential policy implications for developing targeted interventions to mitigate neighborhood deprivation. Future research should delve deeper into the underlying mechanisms driving these disparities and assess the effectiveness of interventions aimed at mitigating them in diverse populations.

Perspectives **COMPETENCY IN MEDICAL KNOWLEDGE:** Our study highlights the significant role of neighborhood deprivation, as measured by ADI, in driving racial disparities in HF outcomes. NHB patients were found to have a higher risk of 1-year HF readmission and mortality compared to their NHW counterparts, even after adjusting for clinical, demographic, and socioeconomic factors. Using a novel counterfactual modeling approach, we demonstrated that improving socioeconomic conditions in deprived neighborhoods could modestly reduce these disparities. These findings emphasize the importance of integrating SDoH into clinical practice to enhance risk stratification, care management, and overall outcomes in HF populations.

**TRANSLATIONAL OUTLOOK:** These findings suggest a need for public health initiatives aimed at addressing the impacts of neighborhood deprivation on racial disparities in HF outcomes. Strategies may include community-based programs to improve socioeconomic conditions, targeted interventions to enhance access to health care resources, and policies designed to address structural inequities in deprived neighborhoods. Public health officials and policymakers should consider incorporating SDoH into care models for HF patients to promote equitable outcomes. Additionally, investments in health care infrastructure and data systems that integrate socioeconomic information can enable proactive identification and support of at-risk populations, fostering resilience in managing chronic conditions like HF.

## Funding support and author disclosures

This work was supported by 10.13039/100001797PhRMA Foundation Research Starter Award (2022 RSG 965016). The authors have reported that they have no relationships relevant to the contents of this paper to disclose.
