# Supplementary material for: Neighborhood Deprivation and Racial Disparities in Heart Failure Outcomes: A Counterfactual Approach
Source: JACC Adv. 2025 Jun 25;4(6):101808. doi: 10.1016/j.jacadv.2025.101808 (PMC12277623; doi:10.1016/j.jacadv.2025.101808)
Supplement: Supplemental Material [file mmc1.docx]

Supplemental Figure 1 Directed Acyclic Graph for heart failure readmissions and mortality


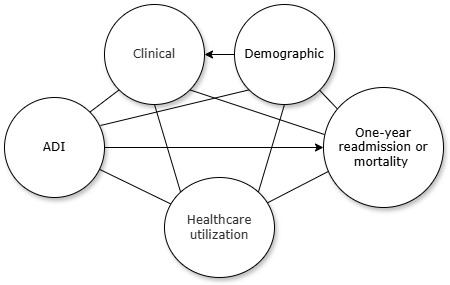


Supplemental Figure 2 DHARMa residual diagnostics assessing model fit.


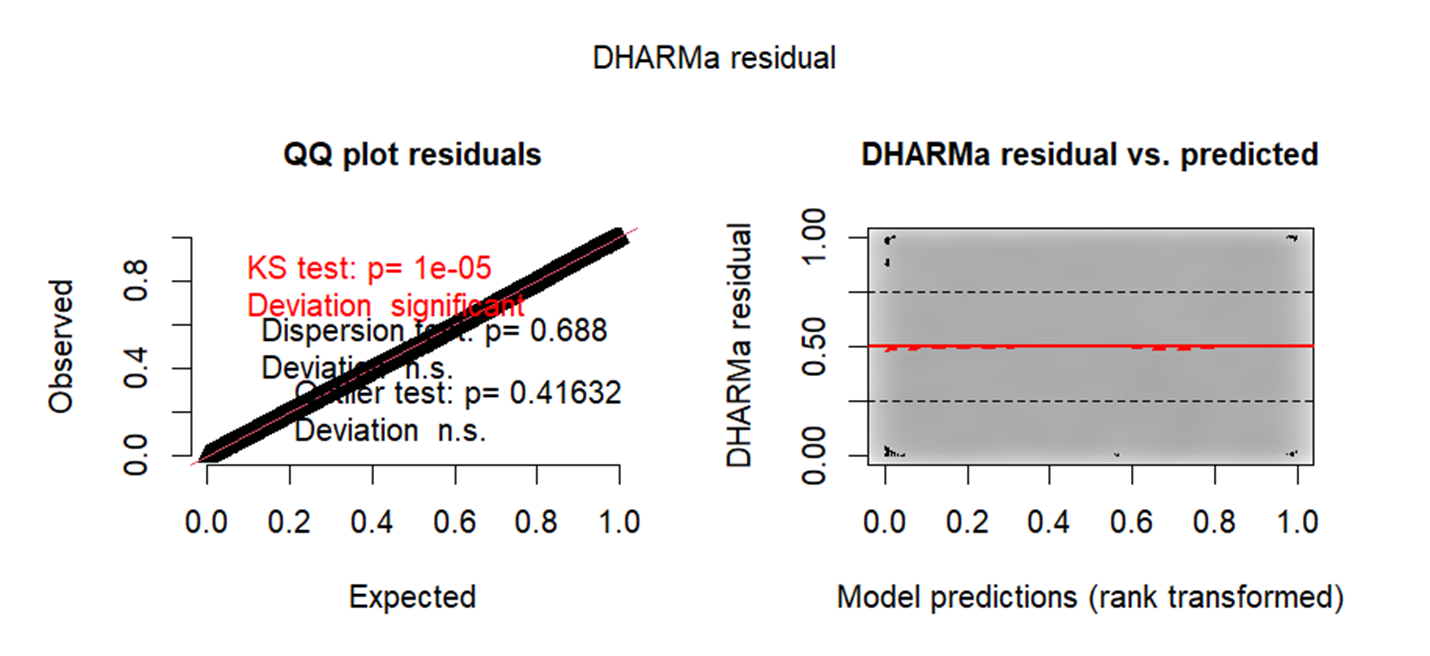


The left panel is a QQ plot of the simulated residuals (Kolmogorov-Smirnov test p=1e-05), indicating a mild departure from uniformity but no significant overdispersion. The right panel is the DHARMa residual vs. predicted plot, showing no major systematic biases.

Supplemental Table 1 Disease identification

| Comorbidities | ICD | Procedure codes |
| --- | --- | --- |
| Heart failure with preserved ejection fraction | ICD-9: 428.3;  ICD-10: I50.3; |  |
| Chronic obstructive pulmonary disease | ICD-9: 490, 491.0, 491.1, 491.8, 491.9, 492.0, 492.8, 491.20, 491.21, 491.22, 494.0, 494.1, 496;  ICD-10: J40, J41.0, J41.1, J41.8, J42, J43.0, J43.1, J43.2, J43.8, J43.9, J44.0, J44.1, J44.9, J47.0, J47.1, J47.9; |  |
| Myocardial infarction | ICD-9: 412;  ICD-10: I252; |  |
| Anemia | ICD-9: 280.0, 280.1, 280.8, 280.9, 281.0, 281.1, 281.2, 281.3, 281.4, 281.8, 281.9, 282.0, 282.1, 282.2, 282.3, 282.40, 282.41, 282.42, 282.43, 282.44, 282.45, 282.46, 282.47, 282.49, 282.5, 282.60, 282.61, 282.62, 282.63, 282.64, 282.68, 282.69, 282.7, 282.8, 282.9, 283.0, 283.10, 283.11, 283.19, 283.2, 283.9, 284.01, 284.09, 284.11, 284.12, 284.19, 284.2, 284.81, 284.89, 284.9, 285.0, 285.1, 285.21, 285.22, 285.29, 285.3, 285.8, 285.9;  ICD-10: D50.0, D50.1, D50.8, D50.9, D51.0, D51.1, D51.2, D51.3, D51.8, D51.9, D52.0, D52.1, D52.8, D52.9, D53.0, D53.1, D53.2, D53.8, D53.9, D55.0, D55.1, D55.2, D55.3, D55.8, D55.9, D56.0, D56.1, D56.2, D56.3, D56.4, D56.5, D56.8, D56.9, D57.00, D57.01, D57.02, D57.1, D57.20, D57.211, D57.212, D57.219, D57.3, D57.40, D57.411, D57.412, D57.419, D57.80, D57.811, D57.812, D57.819, D58.0, D58.1, D58.2, D58.8, D58.9, D59.0, D59.1, D59.2, D59.3, D59.4, D59.5, D59.6, D59.8, D59.9, D60.0, D60.1, D60.8, D60.9, D61.01, D61.09, D61.1, D61.2, D61.3, D61.810, D61.811, D61.818, D61.82, D61.89, D61.9, D62, D63.0, D63.1, D63.8, D64.0, D64.1, D64.2, D64.3, D64.4, D64.81, D64.89, D64.9; |  |
| Implantable cardioverter-defibrillator and/or cardiac resynchronization therapy | ICD-9: V45.0;  ICD-10: Z95.0; | CPT: 33224, 33225, 33226, 33206, 33207, 33208, 33214, 33227, 33228, 33229, 33212, 33213, 33221;  ICD-10-CM: Z95.810, Z95.0;  ICD-9-CM: V45.02, V45.01 |
| Diabetes | ICD-9: 249.00, 249.01, 249.10, 249.11, 249.20, 249.21, 249.30, 249.31, 249.40, 249.41, 249.50, 249.51, 249.60, 249.61, 249.70, 249.71, 249.80, 249.81, 249.90, 249.91, 250.00, 250.01, 250.02, 250.03, 250.10, 250.11, 250.12, 250.13, 250.20, 250.21, 250.22, 250.23, 250.30, 250.31, 250.32, 250.33, 250.40, 250.41, 250.42, 250.43, 250.50, 250.51, 250.52, 250.53, 250.60, 250.61, 250.62, 250.63, 250.70, 250.71, 250.72, 250.73, 250.80, 250.81, 250.82, 250.83, 250.90, 250.91, 250.92, 250.93, 357.2, 362.01, 362.02, 362.03, 362.04, 362.05, 362.06, 366.41;  ICD-10: E08.00, E08.01, E08.10, E08.11, E08.21, E08.22, E08.29, E08.311, E08.319, E08.321, E08.3211, E08.3212, E08.3213, E08.3219, E08.329, E08.3291, E08.3292, E08.3293, E08.3299, E08.331, E08.3311, E08.3312, E08.3313, E08.3319, E08.339, E08.3391, E08.3392, E08.3393, E08.3399, E08.341, E08.3411, E08.3412, E08.3413, E08.3419, E08.349, E08.3491, E08.3492, E08.3493, E08.3499, E08.351, E08.3511, E08.3512, E08.3513, E08.3519, E08.3521, E08.3522, E08.3523, E08.3529, E08.3531, E08.3532, E08.3533, E08.3539, E08.3541, E08.3542, E08.3543, E08.3549, E08.3551, E08.3552, E08.3553, E08.3559, E08.359, E08.3591, E08.3592, E08.3593, E08.3599, E08.36, E08.37X1, E08.37X2, E08.37X3, E08.37X9, E08.39, E08.40, E08.41, E08.42, E08.43, E08.44, E08.49, E08.51, E08.52, E08.59, E08.610, E08.618, E08.620, E08.621, E08.622, E08.628, E08.630, E08.638, E08.641, E08.649, E08.65, E08.69, E08.8, E08.9, E09.00, E09.01, E09.10, E09.11, E09.21, E09.22, E09.29, E09.311, E09.319, E09.321, E09.3211, E09.3212, E09.3213, E09.3219, E09.329, E09.3291, E09.3292, E09.3293, E09.3299, E09.331, E09.3311, E09.3312, E09.3313, E09.3319, E09.339, E09.3391, E09.3392, E09.3393, E09.3399, E09.341, E09.3411, E09.3412, E09.3413, E09.3419, E09.349, E09.3491, E09.3492, E09.3493, E09.3499, E09.351, E09.3511, E09.3512, E09.3513, E09.3519, E09.3521, E09.3522, E09.3523, E09.3529, E09.3531, E09.3532, E09.3533, E09.3539, E09.3541, E09.3542, E09.3543, E09.3549, E09.3551, E09.3552, E09.3553, E09.3559, E09.359, E09.3591, E09.3592, E09.3593, E09.3599, E09.36, E09.37X1, E09.37X2, E09.37X3, E09.37X9, E09.39, E09.40, E09.41, E09.42, E09.43, E09.44, E09.49, E09.51, E09.52, E09.59, E09.610, E09.618, E09.620, E09.621, E09.622, E09.628, E09.630, E09.638, E09.641, E09.649, E09.65, E09.69, E09.8, E09.9, E10.10, E10.11, E10.21, E10.22, E10.29, E10.311, E10.319, E10.321, E10.3211, E10.3212, E10.3213, E10.3219, E10.329, E10.3291, E10.3292, E10.3293, E10.3299, E10.331, E10.3311, E10.3312, E10.3313, E10.3319, E10.339, E10.3391, E10.3392, E10.3393, E10.3399, E10.341, E10.3411, E10.3412, E10.3413, E10.3419, E10.349, E10.3491, E10.3492, E10.3493, E10.3499, E10.351, E10.3511, E10.3512, E10.3513, E10.3519, E10.3521, E10.3522, E10.3523, E10.3529, E10.3531, E10.3532, E10.3533, E10.3539, E10.3541, E10.3542, E10.3543, E10.3549, E10.3551, E10.3552, E10.3553, E10.3559, E10.359, E10.3591, E10.3592, E10.3593, E10.3599, E10.36, E10.37X1, E10.37X2, E10.37X3, E10.37X9, E10.39, E10.40, E10.41, E10.42, E10.43, E10.44, E10.49, E10.51, E10.52, E10.59, E10.610, E10.618, E10.620, E10.621, E10.622, E10.628, E10.630, E10.638, E10.641, E10.649, E10.65, E10.69, E10.8, E10.9, E11.00, E11.01, E11.10, E11.11, E11.21, E11.22, E11.29, E11.311, E11.319, E11.321, E11.3211, E11.3212, E11.3213, E11.3219, E11.329, E11.3291, E11.3292, E11.3293, E11.3299, E11.331, E11.3311, E11.3312, E11.3313, E11.3319, E11.339, E11.3391, E11.3392, E11.3393, E11.3399, E11.341, E11.3411, E11.3412, E11.3413, E11.3419, E11.349, E11.3491, E11.3492, E11.3493, E11.3499, E11.351, E11.3511, E11.3512, E11.3513, E11.3519, E11.3521, E11.3522, E11.3523, E11.3529, E11.3531, E11.3532, E11.3533, E11.3539, E11.3541, E11.3542, E11.3543, E11.3549, E11.3551, E11.3552, E11.3553, E11.3559, E11.359, E11.3591, E11.3592, E11.3593, E11.3599, E11.36, E11.37X1, E11.37X2, E11.37X3, E11.37X9, E11.39, E11.40, E11.41, E11.42, E11.43, E11.44, E11.49, E11.51, E11.52, E11.59, E11.610, E11.618, E11.620, E11.621, E11.622, E11.628, E11.630, E11.638, E11.641, E11.649, E11.65, E11.69, E11.8, E11.9, E13.00, E13.01, E13.10, E13.11, E13.21, E13.22, E13.29, E13.311, E13.319, E13.321, E13.3211, E13.3212, E13.3213, E13.3219, E13.329, E13.3291, E13.3292, E13.3293, E13.3299, E13.331, E13.3311, E13.3312, E13.3313, E13.3319, E13.339, E13.3391, E13.3392, E13.3393, E13.3399, E13.341, E13.3411, E13.3412, E13.3413, E13.3419, E13.349, E13.3491, E13.3492, E13.3493, E13.3499, E13.351, E13.3511, E13.3512, E13.3513, E13.3519, E13.3521, E13.3522, E13.3523, E13.3529, E13.3531, E13.3532, E13.3533, E13.3539, E13.3541, E13.3542, E13.3543, E13.3549, E13.3551, E13.3552, E13.3553, E13.3559, E13.359, E13.3591, E13.3592, E13.3593, E13.3599, E13.36, E13.39, E13.40, E13.41, E13.42, E13.43, E13.44, E13.49, E13.51, E13.52, E13.59, E13.610, E13.618, E13.620, E13.621, E13.622, E13.628, E13.630, E13.638, E13.641, E13.649, E13.65, E13.69, E13.8, E13.9; |  |
| Breast cancer | ICD-9: 174.0, 174.1, 174.2, 174.3, 174.4, 174.5, 174.6, 174.8, 174.9, 175.0, 175.9, 233.0, V10.3;  ICD-10: C50.011, C50.012, C50.019, C50.021, C50.022, C50.029, C50.111, C50.112, C50.119, C50.121, C50.122, C50.129, C50.211, C50.212, C50.219, C50.221, C50.222, C50.229, C50.311, C50.312, C50.319, C50.321, C50.322, C50.329, C50.411, C50.412, C50.419, C50.421, C50.422, C50.429, C50.511, C50.512, C50.519, C50.521, C50.522, C50.529, C50.611, C50.612, C50.619, C50.621, C50.622, C50.629, C50.811, C50.812, C50.819, C50.821, C50.822, C50.829, C50.911, C50.912, C50.919, C50.921, C50.922, C50.929, D05.00, D05.01, D05.02, D05.10, D05.11, D05.12, D05.80, D05.81, D05.82, D05.90, D05.91, D05.92, Z85.3; |  |
| Colorectal cancer | ICD-9: 153.0, 153.1, 153.2, 153.3, 153.4, 153.5, 153.6, 153.7, 153.8, 153.9,154.0,154.1, 230.3, 230.4, V10.05, V10.06;  ICD-10: C18.0, C18.1, C18.2, C18.3, C18.4, C18.5, C18.6, C18.7, C18.8, C18.9, C19, C20, D01.0, D01.1, D01.2, Z85.038, Z85.040, Z85.048; |  |
| Prostate cancer | ICD-9: 185, 233.4, V10.46;  ICD-10: C61, D07.5, Z85.46; |  |
| Lung cancer | ICD-9: 162.2, 162.3, 162.4, 162.5, 162.8, 162.9, 231.2, V10.11;  ICD-10: C34.00, C34.01, C34.02, C34.10, C34.11, C34.12, C34.2, C34.30, C34.31, C34.32, C34.80, C34.81, C34.82, C34.90, C34.91, C34.92, D02.20, D02.21, D02.22, Z85.110, Z85.118; |  |
| Endometrial cancer | ICD-9: 182.0, 233.2, V10.42;  ICD-10: C54.1, C54.2, C54.3, C54.8, C54.9, D07.0, Z85.42; |  |

Supplemental Table 2. Logistic regression model for heart failure readmissions and mortality examining racial differences in the outcome.

| Variable | Estimate | Lower CI | Upper CI |
| --- | --- | --- | --- |
| Intercept | 0.417 | 0.323 | 0.539 |
| Medium ADI | 1.068 | 0.994 | 1.148 |
| High ADI | 1.044 | 0.96 | 1.135 |
| Age: 45-54 years | 0.963 | 0.903 | 1.026 |
| Age: 55-64 years | 0.889 | 0.831 | 0.951 |
| Age: 65+ years | 0.897 | 0.84 | 0.958 |
| Race/Ethnicity: NHB | 1.313 | 1.252 | 1.377 |
| Sex: Male | 1.126 | 1.075 | 1.178 |
| History of HFpEF | 1.076 | 1.027 | 1.128 |
| History of COPD | 1.19 | 1.135 | 1.248 |
| History of MI | 1.137 | 1.072 | 1.207 |
| History of Anemia | 1.111 | 1.059 | 1.165 |
| History of ICD/CRT-D | 1.522 | 1.406 | 1.647 |
| History of Diabetes | 1.175 | 1.12 | 1.232 |
| History of Cancer | 1.16 | 1.084 | 1.241 |

Supplemental Table 3. Logistic regression model for heart failure readmissions and mortality examining race/ethnicity differences in the outcome.

| Variable | Estimate | Lower CI | Upper CI |
| --- | --- | --- | --- |
| Intercept | 0.434 | 0.337 | 0.560 |
| Age: 45-54 years | 0.963 | 0.904 | 1.026 |
| Age: 55-64 years | 0.888 | 0.831 | 0.950 |
| Age: 65+ years | 0.897 | 0.841 | 0.957 |
| Race/Ethnicity: NHB | 1.314 | 1.254 | 1.378 |
| Sex: Male | 1.128 | 1.078 | 1.181 |
| History of HFpEF | 1.075 | 1.026 | 1.126 |
| History of COPD | 1.191 | 1.135 | 1.248 |
| History of MI | 1.135 | 1.069 | 1.204 |
| History of Anemia | 1.111 | 1.060 | 1.166 |
| History of ICD/CRT-D | 1.522 | 1.407 | 1.647 |
| History of Diabetes | 1.174 | 1.120 | 1.231 |
| History of Cancer | 1.163 | 1.087 | 1.245 |

Supplemental Table 4. Logistic regression model for heart failure readmissions and mortality examining ADI differences in the outcome.

| Variable | Estimate | Lower CI | Upper CI |
| --- | --- | --- | --- |
| Intercept | 0.533 | 0.408 | 0.697 |
| Medium ADI | 0.968 | 0.894 | 1.048 |
| High ADI | 0.919 | 0.841 | 1.004 |
| Age: 45-54 years | 0.933 | 0.876 | 0.994 |
| Age: 55-64 years | 0.842 | 0.788 | 0.900 |
| Age: 65+ years | 0.828 | 0.777 | 0.883 |
| Sex: Male | 1.108 | 1.059 | 1.160 |
| History of HFpEF | 1.069 | 1.021 | 1.120 |
| History of COPD | 1.155 | 1.102 | 1.211 |
| History of MI | 1.113 | 1.049 | 1.180 |
| History of Anemia | 1.130 | 1.078 | 1.185 |
| History of ICD/CRT-D | 1.518 | 1.403 | 1.642 |
| History of Diabetes | 1.201 | 1.145 | 1.259 |
| History of Cancer | 1.164 | 1.088 | 1.245 |

Supplemental Table 5. Variance Inflation Factors for Fixed-Effect Covariates

|  | GVIF | Df | GVIF^(1/(2*Df)) |
| --- | --- | --- | --- |
| ADI_NATRANK_q | 1.008831 | 2 | 1.0022 |
| factor(ageg) | 1.087717 | 3 | 1.014112 |
| factor(SEX) | 1.071764 | 1 | 1.03526 |
| factor(HFpEF) | 1.116305 | 1 | 1.056553 |
| factor(COPD_flag) | 1.125083 | 1 | 1.060699 |
| factor(History_of_myocardial_infarction_flag) | 1.107377 | 1 | 1.05232 |
| factor(Anemia_flag) | 1.165412 | 1 | 1.079543 |
| factor(ICD_andor_CRT_D_flag) | 1.077812 | 1 | 1.038177 |
| factor(Diabetes_flag) | 1.150108 | 1 | 1.072431 |
| factor(cancer) | 1.04029 | 1 | 1.019946 |

Supplemental Table 6.

|  | R2m | R2c |
| --- | --- | --- |
| theoretical | 0.016839 | 0.040897 |
| delta | 0.013495 | 0.032777 |

Supplemental Table 7. STROBE Checklist

| **Item** | **Description** | **Page/Section** |
| --- | --- | --- |
| Title and abstract | Clear indication of study design (cohort), objectives, and results | Title page, Abstract |
| Background/rationale | Scientific rationale provided | Introduction |
| Objectives | Clearly stated objectives or hypothesis | Introduction |
| Study design | Cohort design clearly described | Methods |
| Setting | Setting, locations, and study dates described | Methods |
| Participants | Eligibility criteria, exclusion criteria, and sources/methods of selection | Methods, Figure 1 |
| Variables | Clearly defined outcomes, exposures, predictors, potential confounders | Methods |
| Data sources/measurement | Details provided on data sources, data linkage, and measurement methods | Methods |
| Bias | Efforts taken to address potential bias described | Methods |
| Study size | Description of sample size and power | Methods |
| Quantitative variables | Explanation of variable categorization | Methods |
| Statistical methods | Methods detailed, including subgroup analyses, sensitivity analyses, and handling missing data | Methods |
| Participants | Flow of participants detailed | Results, Figure 1 |
| Descriptive data | Baseline characteristics summarized | Table 1 |
| Outcome data | Outcome data clearly reported | Results |
| Main results | Main results presented with confidence intervals | Results |
| Other analyses | Subgroup analyses, sensitivity analyses reported | Results |
| Key results | Key results summarized | Discussion |
| Limitations | Study limitations explicitly discussed | Discussion |
| Interpretation | Generalizability of findings discussed | Discussion |
| Funding | Sources of funding disclosed | Title page |

**Additional methodologic detail for the counterfactual modeling**

We built our counterfactual prediction model using a federated algorithm for generalized linear mixed models (Fed-GLMM), which enables the joint implementation of GLMMs using data from multiple sites by leveraging local computations and shared summary statistics^1^. This algorithm can be iteratively performed to refine model parameters by constructing a quadratic surrogate function that approximates the global likelihood function, ensuring robust and accurate modeling in a federated learning environment^2^.

The Fed-GLMM approach consists of three key steps. In the first step, each site fits its own GLMM to obtain initial parameter estimates. In the second step, each site calculates and broadcasts summary statistics, which encapsulate essential information about the local likelihood function and are shared with the central analytics unit. In the third step, the central unit uses these summary statistics to construct a quadratic surrogate global likelihood function, which is then used to update the parameter estimates. This iterative process ensures continuous improvement in model accuracy while safeguarding data privacy across the participating sites.

In our simulation analysis, we assigned each non-Hispanic Black (NHB) patient to one of three ADI (Area Deprivation Index) regions using a multinomial distribution. The probabilities for this distribution were derived from the proportions of non-Hispanic White (NHW)patients in these regions. We then estimated the individual risk of one-year readmission or death for NHB patients as if they resided in their assigned ADI region. The mean of these estimated patient-level risks was used to determine the overall risk for the population based on the new ADI region assignments. This procedure was repeated 400 times to obtain estimates of uncertainty.

References

1. Tong J, Shen Y, Xu A, et al. Evaluating site-of-care-related racial disparities in kidney graft failure using a novel federated learning framework. *J Am Med Inform Assoc*. 2024;31(6):1303-1312. doi:10.1093/jamia/ocae075

2. Yan Z, Zachrison KS, Schwamm LH, Estrada JJ, Duan R. A privacy-preserving and computation-efficient federated algorithm for generalized linear mixed models to analyze correlated electronic health records data. *PLoS One*. 2023;18(1):e0280192. doi:10.1371/journal.pone.0280192
